# Isolated meniscal repair, medial meniscus repair and older age increase failure risk after all‐inside meniscal repair: A cohort study of 2264 patients

**DOI:** 10.1002/ksa.12780

**Published:** 2025-07-07

**Authors:** Christoffer von Essen, Riccardo Cristiani, Carolina Kekki, Dzan Rizvanovic, Anders Stålman

**Affiliations:** ^1^ Department of Molecular Medicine and Surgery, Section of Sports Medicine Karolinska Institutet Stockholm Sweden; ^2^ Stockholm Sports Trauma Research Center (SSTRC), FIFA Medical Centre of Excellence Stockholm Sweden

**Keywords:** age, all‐inside, failure, meniscal repair

## Abstract

**Purpose:**

To evaluate the overall failure rate and assess factors influencing the failure of modern all‐inside meniscal repair.

**Methods:**

Patients who underwent all‐inside meniscal repair at Capio Artro Clinic, Stockholm, Sweden, from January 2015 to June 2022, were identified. The primary outcome was the occurrence of failure of the repair, defined as reoperation and secondary partial or total meniscal resection within three years. Kaplan–Meier analysis was performed to assess meniscal repair survival, with multivariate Cox regression analysis to adjust for confounders.

**Results:**

A total of 2264 patients who underwent meniscal repairs were included (55.9% males, mean age of 27 years ±10). The meniscal repair failure rate for the entire cohort was 20.2% (457/2264). Older age (>40 years) (*p* = 0.03), medial meniscus repair (*p* < 0.001) and isolated meniscal repair (without concomitant anterior cruciate ligament reconstruction [ACLR]) (*p* < 0.001) were associated with statistically significant increased failure rates. Multivariate Cox regression analysis revealed that failure after meniscal repair was statistically significant related to age > 40 (hazard ratio [HR] 1.22; 95% confidence interval (CI) 1.03–1.56; *p* = 0.034), medial meniscus repair (HR 2.57; 95% CI 2.06–3.19; *p* < 0.001) and isolated repairs (HR 2.63; 95% CI 2.17–3.19; *p* < 0.001).

**Conclusions:**

In this large cohort study of more than 2000 patients, the overall failure rate of all‐inside meniscal repair was 20.2%. Repair without concomitant ACLR, medial meniscal repair, and older age were associated with an increased hazard of failure. These findings underscore the clinical importance of recognising patient‐specific risk factors for meniscal repair failure, as such knowledge can guide surgical decision‐making and improve individualised treatment strategies to enhance long‐term knee function.

**Level of Evidence:**

Level III.

**Abbreviations**
ACL: anterior cruciate ligament
ACLR: anterior cruciate ligament reconstruction
CI: confidence interval
HR: hazard ratio
ICD‐10: International Classification of Diseases, 10th Revision
KOOS: Knee injury and Osteoarthritis Outcome Score
MRI: magnetic resonance imaging
ROM: range of motion
SD: standard deviation

## INTRODUCTION

Meniscal surgeries are among the most frequently performed procedures in orthopaedic practice, reflecting the critical role of the meniscus in maintaining knee joint mechanics and stability [1, 2, 12]. The menisci serve as shock absorbers, distribute compressive loads, and contribute to knee stabilisation during complex movements and weight‐bearing activities [36]. Damage to the menisci, especially in combination with anterior cruciate ligament (ACL) injuries, has significant implications for knee laxity and long‐term joint health, potentially accelerating osteoarthritis [8, 22, 33, 36]. As a result, surgical management has increasingly shifted toward meniscal repair, emphasising the preservation of meniscal function to delay the onset of osteoarthritic changes [22].

The inside‐out technique has long been regarded as the 'gold standard' for meniscal repair due to its consistent outcomes and durability. Failure rates for inside‐out repairs are reported to be up to 25%, reflecting its reliability as a surgical approach [10, 34]. In contrast, the development of all‐inside devices has introduced a paradigm shift, offering practical advantages such as reduced surgical morbidity, shorter operative times, and simpler technical application, making it an appealing option for both surgeons and patients [34].

However, despite these advantages, the reported outcomes for all‐inside meniscal repair remain inconsistent. A recent meta‐analysis highlighted a significantly lower failure rate for inside‐out meniscal repair (5.6%) compared to all‐inside repair (22.3%) [29]. Another meta‐analysis, involving 3829 patients, found an overall failure rate for meniscal repair of 14.8% but reported no significant difference between pooled failure rates for inside‐out and all‐inside techniques (11.9% vs. 10.6%; *p* > 0.05) [28]. These variations highlight the need for further investigation, particularly as newer all‐inside implants have been introduced with improved biomechanical properties.

Despite the growing adoption of all‐inside techniques, most studies in the literature either mix data from inside‐out and all‐inside repairs or include small cohorts, making it difficult to isolate the true performance of modern all‐inside devices [6, 18, 24, 28, 29, 30, 35]. Additionally, the range of failure rates for all‐inside repairs (reported between 5.6% and 22.3%) underscores the variability influenced by differences in surgical expertise, patient populations, and implant design.

This study aimed to investigate failure rates and factors associated with failure of all‐inside meniscal repair in a large cohort. It was hypothesised that modern all‐inside meniscal repair would have an acceptable failure rate and that age, medial vs. lateral meniscus, and concomitant ACL reconstruction (ACLR) would have equivalent failure rates and would not affect the hazard of meniscal failure.

## METHODS

Ethical approval for this study was obtained from the regional ethics committee (Karolinska Institutet, DNR 2024‐06048‐01).

Patients who underwent arthroscopic all‐inside meniscal repair from January 2015 to June 2022 at Capio Artro Clinic, Stockholm, Sweden, were identified retrospectively using procedural (ICD‐10) codes, m232, S832, S837. Surgical and medical journals were reviewed, and patient characteristics were collected.

Inclusion criteria were as follows: (1) medial or lateral all‐inside meniscal repair, (2) isolated meniscal repair or meniscal repair in combination with ACLR and (3) no previous meniscal surgery in the index knee. Patients were excluded if the meniscal repair was concomitant with a multiligamentous injury, or if there was an intra‐articular fracture. All inside‐out or outside‐in techniques were excluded, as well as isolated root repairs due to their complexity.

The primary study endpoint was the failure of meniscal repair. Failure was defined as reoperation with a partial or total meniscal resection within 3 years on the previous sutured meniscus. Previous studies have shown that the majority of meniscal repair failures occur within the first 2 years postoperatively, with diminishing rates thereafter. By extending the follow‐up to 3 years, we aimed to capture both early and intermediate‐term failures while maintaining a robust and reliable dataset. However, it was not confirmed if the reoperation was due to new pathology or a clear failure.

### Surgical technique

All operations were undertaken by consultant knee surgeons, all specialising in arthroscopic knee surgery. Overall, 22 high‐volume sports medicine surgeons performed the procedures [26].

Standard arthroscopic anteromedial and anterolateral portals were utilised, and all tears underwent preparation in the form of rasping the tear site and adjacent synovium, followed by anatomic reduction. Repairs were performed arthroscopically using all‐inside devices (FasT‐fix360 (Smith & Nephew, USA) or FiberStitch (Arthrex, USA). Sutures were placed in vertical, horizontal, or oblique configurations on the superior and inferior surfaces of the meniscus, depending on the tear configuration and surgeon's assessment. The number of sutures used varied based on the requirement to achieve a rigid and stable construct. ACLRs were all performed with a single‐bundle technique with either hamstring tendons, bone‐patellar tendon‐bone or quadriceps tendon autografts using suspensory femoral fixation (Endobutton CL, Smith & Nephew, USA, or Tightrope, Arthrex, USA). The femoral tunnel was drilled via the anteromedial portal and standard tibial fixation with an AO screw as a washer or an interference screw.

A standardised rehabilitation protocol was used, where full weight‐bearing was allowed from Day 1, except for radial tears, where no weight‐bearing was allowed for 6 weeks. All patients wore a hinged brace with a fixed range of motion (ROM) for a total of 6 weeks (0°–30°, 0°–60° and 0°–90° with unlocking every two weeks) and were instructed to avoid squatting for a total of 12–16 weeks. For patients with an isolated meniscal repair, no return to pivoting sports was recommended before 4 months, and for patients with a concomitant ACLR, before 9 months.

### Statistical analysis

Statistical analysis was performed with the SPSS (version 25.0, IBM Corp., NY, USA) software package. Continuous variables were described as the mean (standard deviation [SD]) and categorical variables with count (*n*) and proportions (%). Comparisons between the failed and non‐failed cohorts were performed with an independent Student's *t*‐test for continuous variables and Pearson's chi‐square test for categorical variables. To account for multiple comparisons in subgroup analyses in age groups, a Bonferroni correction was applied. The significance threshold was adjusted accordingly and made to reduce the risk of Type 1 error. Meniscal repair survival was assessed with Kaplan–Meier analysis. A Cox regression analysis was used to investigate factors associated with meniscal repair failure. Results were expressed as hazard ratios (HRs) with 95% CI. *p* < 0.05 was considered statistically significant.

## RESULTS

A total of 2264 patients who underwent meniscal repair were included. The mean age was 27 years ( ± 10) and 55.9% were men. From this cohort 20.2% (457/2264) were classified as failure due to a partial or total resection on the repaired meniscus within 3 years of surgery. Patient characteristics are reported in Table 1.

**Table 1:** Patient characteristics.

	Total	Meniscus repair failure		*p*‐Value
		Yes	No	
No. patients	2264	457 (20.2)	1807 (79.8)	
Age, years				
Mean (SD)	27 (10)	27 (10)	26 (10)	
<40	1941 (85.7)	378 (19.5)	1563 (80.5)	0.03
≥40	323 (14.3)	79 (24.5)	244 (75.5)	
<20	856 (37.8)	160 (18.7)	696 (81.3)	
21–30	680 (30.0)	141 (20.7)	539 (79.3)	
31–39	405 (17.9)	77 (19.0)	328 (81.0)	
≥40	323 (14.3)	79 (24.5)	244 (75.5)	<0.01*
Gender				
Female	999 (44.1)	219 (21.9)	280 (78.1)	n.s.
Male	1265 (55.9)	238 (18.8)	1027 (81.2)	
Meniscus				
Medial	1330 (58.7)	352 (26.5)	978 (73.5)	<0.01
Lateral	934 (41.3)	105 (11.2)	839 (88.8)	
ACL				
No ACL injury	1008 (44.5)	296 (29.4)	712 (70.6)	<0.01
Concomitant ACLR	1256 (55.5)	161 (12.8)	1095 (87.2)	

*Note*: Data are reported as *n* (%) unless otherwise indicated.

Abbreviations: ACL, anterior cruciate ligament; ACLR, anterior cruciate ligament reconstruction; SD, standard deviation.

* Bonferroni‐adjusted.

The majority of failures (79.5% 344/457) occurred within the first 2 postoperative years, and the mean time to failure was 13.7 (9.3) months. The mean time to failure was significantly shorter in patients older than 40 years (9.5 vs. 14.9; *p* < 0.01) and for meniscal repairs performed in conjunction with ACLR (13.2 vs. 15.3; *p* = 0.01), Table 2.

**Table 2:** Mean time to failure.

	Mean time to failure (months [SD])	*p*‐Value
	13.7 (9.3)	
Age, years		
Mean (SD)		
<40	14.9 (9.1)	<0.01
≥40	9.5 (9.3)	
<20	16.2 (9.1)	
21–30	13.9 (9.3)	
31–39	13.7 (8.4)	
≥40	9.5 (9.3)	<0.01*
Gender		
Female	13.8 (9.3)	n.s.
Male	14.0 (9.4)	
Meniscus		
Medial	14.0 (9.3)	n.s.
Lateral	13.8 (9.4)	
ACL		
No ACL injury	13.2 (9.2)	0.01
Concomitant ACLR	15.3 (9.4)	

*Note*: Data are reported as *n* (%) unless otherwise indicated.

Abbreviations: ACL, anterior cruciate ligament; ACLR, anterior cruciate ligament reconstruction; SD, standard deviation.

* Bonferroni‐adjusted.

**Figure 1:**
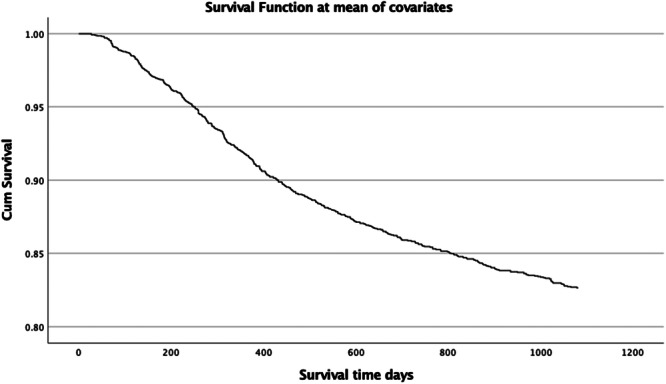
Kaplan–Meier survival analysis shows the probability of meniscal survival over time in days. A greater decrease in survival within the first 2 postoperative years compared with the third postoperative year can be observed.

Analysis of meniscal repair survival using a Kaplan–Meier curve shows a greater decrease in survival within the first 2 postoperative years compared with the third postoperative year, Figure 1.

**Figure 2:**
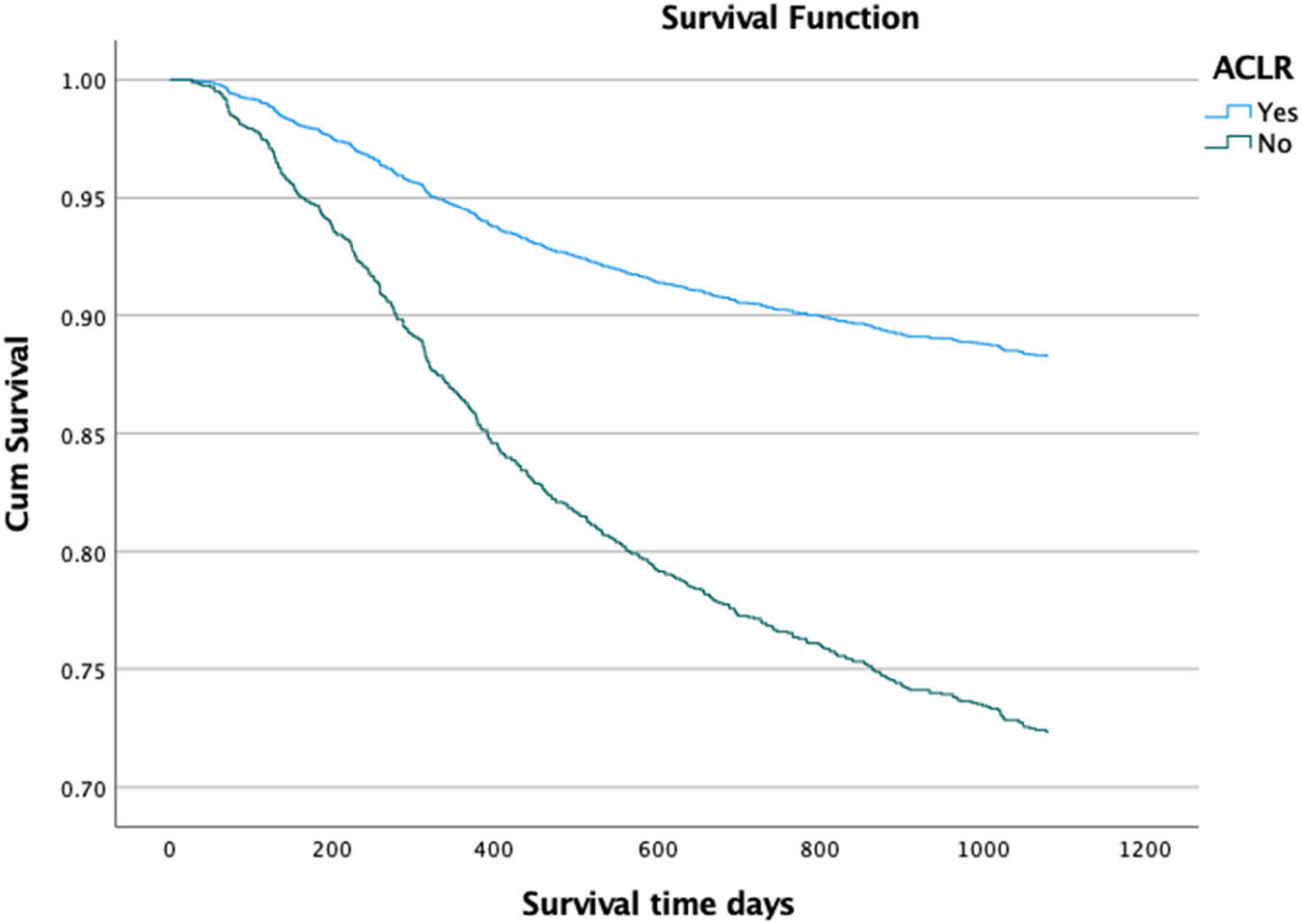
Kaplan–Meier survival analysis shows the probability of survival over time in days for isolated meniscal repair and meniscal repair performed in conjunction with anterior cruciate ligament reconstruction.

Meniscal repair performed in conjunction with ACLR demonstrated lower failure rates than isolated meniscal repair (12.8% vs. 29.4%, respectively; *p* < 0.01). Kaplan–Meier survival analysis is presented in Figure 2.

**Figure 3:**
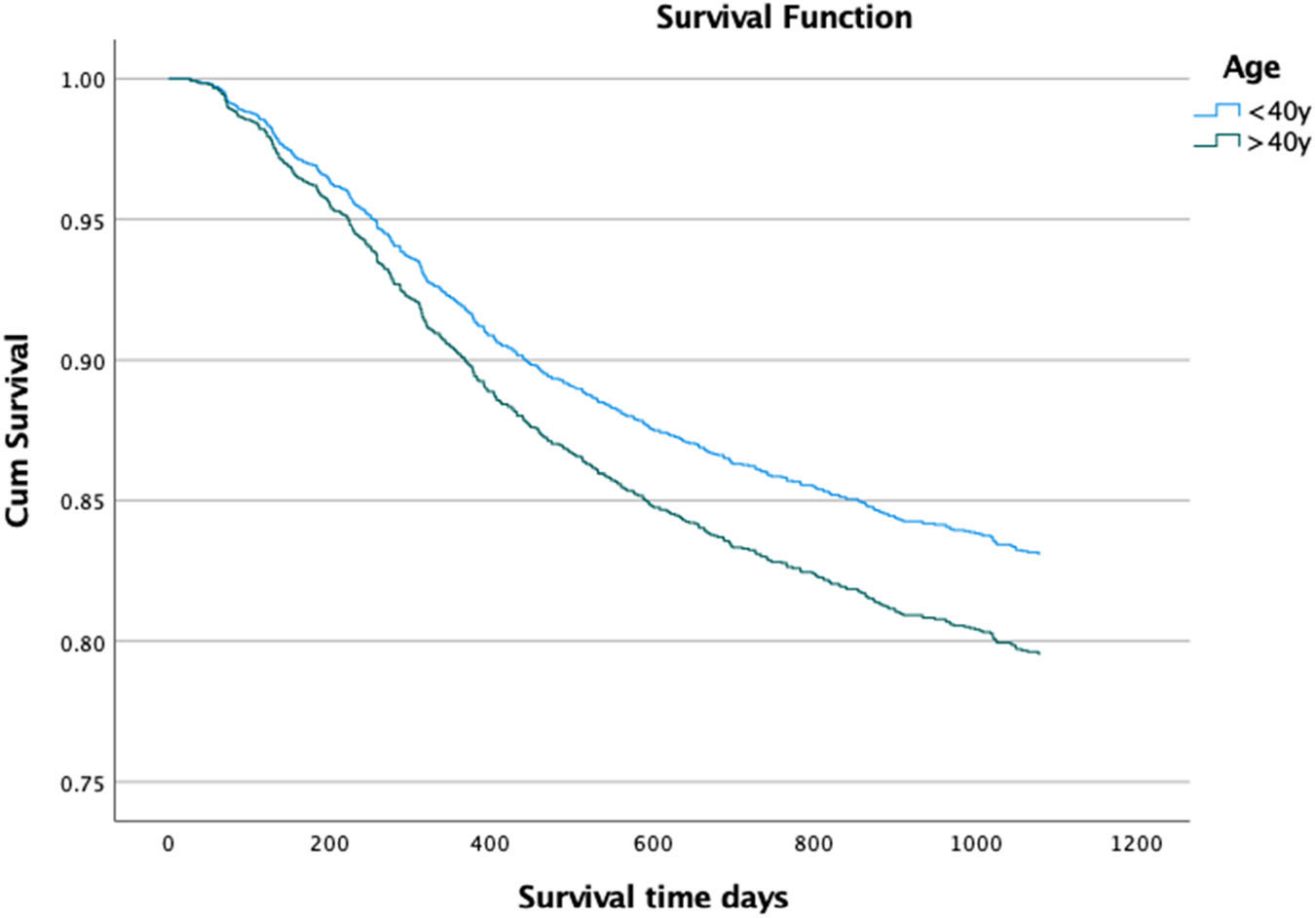
Kaplan–Meier survival analysis shows the probability of meniscal repair survival over time in days depending on age (<40 vs. ≥40 years).

Meniscal repair in patients older than 40 years of age resulted in a significantly higher rate of failure compared to younger (<40 years) patients (24.5% vs. 19.5%, respectively; *p* = 0.03) (Table 1). Kaplan–Meier survival analysis is presented in Figure 3.

**Figure 4:**
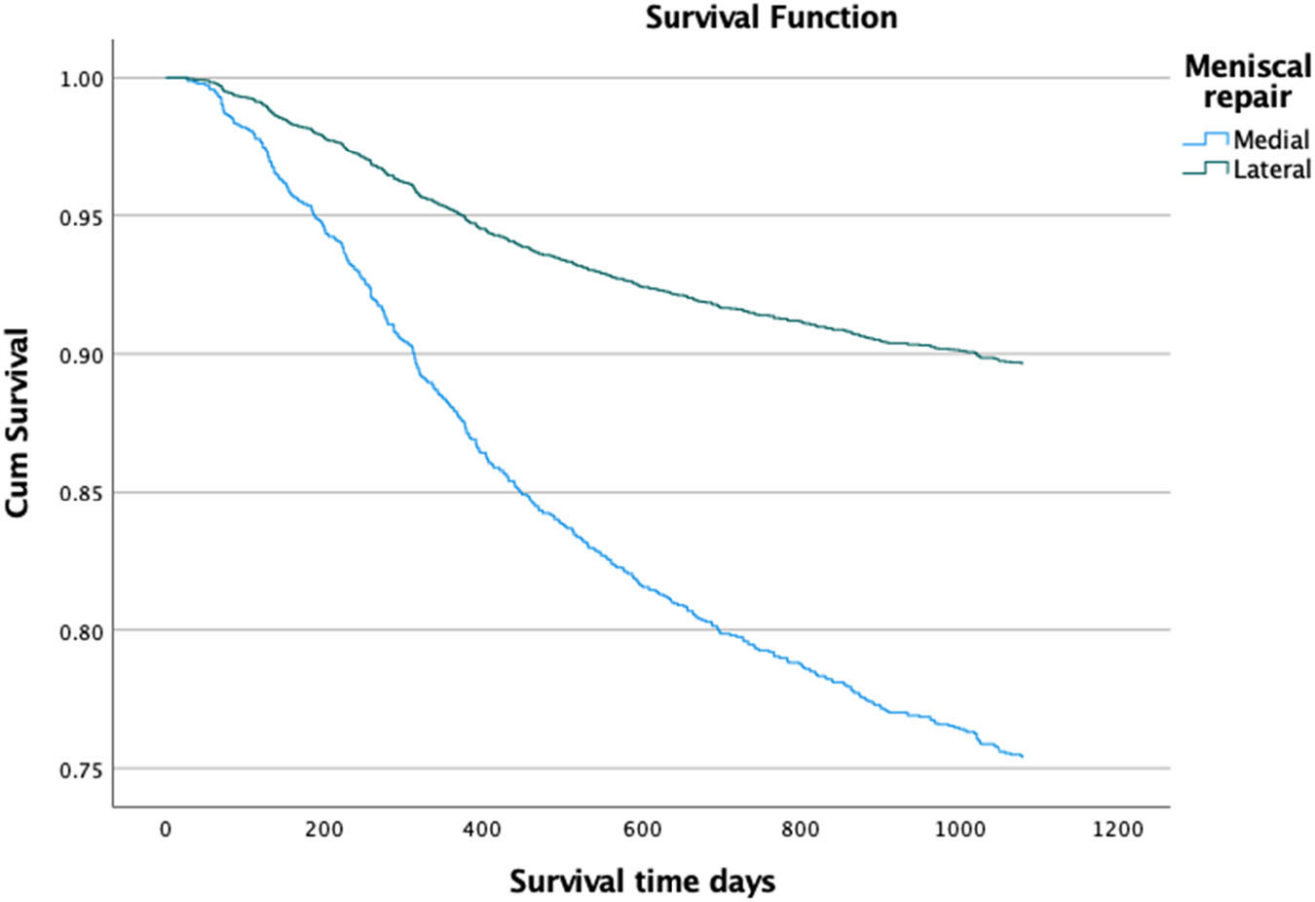
Kaplan–Meier survival analysis shows the probability of survival over time in days for medial and lateral meniscal repair.

Medial meniscal repairs had significantly higher failure rates within 3 years than lateral meniscal repairs (26.5% vs. 11.2%, respectively; *p* < 0.01) (Table 1). Kaplan–Meier survival analysis is presented in Figure 4.

The multivariate Cox regression analysis showed that older age (>40 years), medial meniscal repair, and isolated meniscal repair (without concomitant ACLR) were associated with an increased hazard of failure within 3 years (Table 3).

**Table 3:** Factors associated with failure of meniscal repair in Cox regression analysis.

Factor	Group	Univariate Cox regression		*p*‐Value	Multivariate Cox regression		*p*‐Value
		HR	95% CIs		HR	95% CIs	
Age	<40	1.00 (Reference)		1.00 (Reference)			
	≥40	1.34	1.05–1.71	0.018	1.22	1.03–1.56	0.034
Sex	Male	1.00 (Reference)		1.00 (Reference)			
	Female	1.22	1.02–1.47	0.032	1.19	0.99–1.42	n.s.
Meniscus	Lateral	1.00 (Reference)		1.00 (Reference)			
	Medial	2.56	2.06–3.19	<0.001	2.57	2.06–3.19	<0.001
ACL	ACLR	1.00 (Reference)		1.00 (Reference)			
	No ACLR	2.57	2.12–3.11	<0.001	2.63	2.17–3.19	<0.001

Abbreviations: ACL, anterior cruciate ligament; ACLR, anterior cruciate ligament reconstruction; CI, confidence interval; HR, hazard ratio.

## DISCUSSION

The most important finding of this study was that the overall failure rate for all‐inside meniscal repair was 20.2% at three years postoperatively. Higher age (>40 years), medial meniscal repair, and isolated meniscal repair (no concomitant ACLR) were significantly associated with an increased risk of failure. Additionally, the time to failure was shorter in older patients (≥40 years), and most failures (79.5%) occurred within the first two postoperative years. These findings are important for understanding the prognosis and risks of meniscal repair, aiding in patient counselling, and for informed decision‐making.

The failure rate is one of the key outcomes for judging the results of meniscal repair. To adequately address the failure rate, the definition of failure needs to be defined. The most common definition of failure is the need for subsequent partial or total meniscectomy [27, 29], which is the definition in the present study as well. However, it is not stated if the reoperation of the meniscus is performed in the same location as the previous tear, or if the meniscus has partially healed. Magnetic resonance imaging (MRI) could be an option to assess meniscus healing. However, signal changes in the meniscus can persist for a long time and changes can also be seen in asymptomatic knees, which could make it difficult to differentiate between an injury and normal postoperative changes [37]. A deeper analysis of this would be of value to better understand the pathomechanism of re‐tears.

Traditionally, the inside‐out technique has been the gold standard for meniscal repair; but the all‐inside technique has gained popularity in recent years [28]. All‐inside devices can be differentiated between early‐generation and modern‐generation, where the early‐generation devices, such as meniscal arrows, showed higher failure rates and complications [14, 15]. New generation all‐inside devices are one‐handed with all‐suture anchors, have different angles for anatomical repair, and less traumatic needles. Nepple et al. [18] compared long‐term outcomes between new and early‐generation all‐inside devices and confirmed that early‐generation all‐inside devices had a significantly higher failure rate. Furthermore, several meta‐analyses comparing all‐inside and inside‐out meniscal repair techniques showed no difference in failure rate; however, all‐inside repair was associated with a significantly shorter operating time [4, 28, 35].

The present study found a meniscal repair failure rate of 20.2% for the new generation of all‐inside devices, which is similar to other studies [18, 27, 28, 29].

In this analysis, 79.5% of all meniscal repair failures occurred within the first 24 months. In a recent systematic review by Schweizer et al. [28], 86% of all failures occurred within the first 2 years. On the other hand, Ow et al. [21] reported an increasing failure rate with time. One possible explanation for the high rate of failures occurring within the first 2 years is that this period represents the critical window for biological healing. During this time, patients typically resume sport activities, which may place increased stress on the meniscus. Additionally, early failures may reflect inadequate biological healing due to poor vascular supply, especially in older patients.

There is not sufficient data in this analysis to state whether there is a re‐tear of the repaired meniscus or a new tear adjacent to the repair. The resumption of previous sports activities may cause the repair to fail or a new tear to arise.

This study demonstrated a significant difference in failure rates of medial and lateral meniscus repairs, as well as a significant HR in favour of lateral meniscus repair. A recent meta‐analysis by Schweizer et al. [29] found no difference in the pooled failure rate between lateral and medial repair. In contrast, other systematic reviews, with larger sample sizes, showed a significantly higher failure rate for medial repair. The medial meniscus anatomically is more fixed to the plateau and medial collateral ligament and biomechanically exposed to more force [23]. The medial meniscus also exhibits inferior vascularisation [20]. This may contribute to the superior healing of the lateral meniscus.

A rupture of the ACL often occurs in combination with a meniscal tear and concomitant ACLR resulting in significantly lower meniscal repair failure rates in this study (12.8% vs. 29.4%, respectively; *p* < 0.01). Similar findings have been reported in previous studies [9, 28]. There are several potential reasons for this. One hypothesis is that the release of growth factors and blood during ACLR may enhance the healing of the repair [13]. Another potential reason might be that patients follow a slower and less aggressive rehabilitation protocol after ACLR.

There have been concerns about repairing meniscal tears in older patients, as they tend to be more degenerative. Several studies have tried to identify age as a risk factor that affects failure but have not found any difference in failure rates depending on age [3, 5]. Patient‐reported outcomes after meniscal repair or meniscectomy in patients ≥ 40 years have been reported to be similar [6, 7, 25]. Steadman et al. [32] compared patient satisfaction and failure rates of meniscal repair between patients younger and older than 40 years and could not find any difference. Similarly, the 2019 ESSKA consensus statement on the management of traumatic meniscus tears stated that age does not appear to affect meniscal repair failure rates [13]. Everhart et al. [6] published a systematic review on meniscal repair outcomes in patients aged 40 years and older. They reviewed 11 studies and 148 patients and concluded that failure rates in this age category were comparable to those of younger patients. Similar results were found in another systematic review [30]. In contrast, this study found a significantly higher failure rate and HR for patients 40 years or older compared with patients <40 years (19.5% vs. 24.5%, respectively; *p* = 0.03, HR: 1.22; 95% CI 1.03–1.56; *p* = 0.034), something that has not been shown in previous studies. This discrepancy may be attributed to several factors. First, this could be due to the reduced vascularity in the meniscus and degenerative changes that come with age [16]. Second, the exclusive use of modern all‐inside repair devices in this study may yield different outcomes compared to earlier studies that included older‐generation implants or mixed techniques. These newer devices offer improved biomechanical properties and may therefore be superior. Additionally, previous studies may also suffer from selection bias, as surgeons may attempt a repair in older patients only if there is a high chance of success, whereas in younger patients, a repair is often pursued at any cost. The cohort included in the present study is larger than those of previous systematic reviews, making it less sensitive to underpower. Although meniscal repair is less successful in older patients, age should not be the only factor for the decision of meniscal repair.

Younger patients are more likely to undergo meniscal repair [3]. Other studies [11, 19] have reported a higher incidence of failure with retear rates greater than 30% at 10 years in the adolescent population, this could not be seen in the current study [17].

Younger patients may have an increased risk of failure and have a relatively greater incidence of bucket‐handle tears [31]. Furthermore, a younger population will likely return to sports at a higher rate than their adult counterpart. As the postoperative follow‐up time in this study was limited to three years, the higher retear rates reported in other studies might reflect new injuries rather than failures of the original repair. Ow et al. [21] reported that failure rates increased when younger patients returned to sport.

The main strength of this study was the analysis of a large cohort, with a thorough comprehensive analysis of the medical records to ensure that all the reoperations were included. Moreover, the patients underwent surgery at a large volume clinic specialised in arthroscopic knee surgery and meniscal repair, which may affect the generalisability of the study. Another strength was the specific inclusion of patients undergoing all‐inside meniscal repair, in contrast to other studies that included heterogeneous repair techniques such as inside‐out and outside‐in repairs.

This study has limitations; one is the retrospective design. Additionally, there was limited information regarding meniscal tear patterns as well as patient characteristics such as body mass index. Furthermore, the aetiology (traumatic/degenerative) was not specified in the charts, as well as the time from the potential injury to surgery. Another limitation is that the cause of failure was not documented, making it unclear whether the failure was due to a failed repair, a tear adjacent to the previous repair, or a new tear leading to subsequent surgery. It is also possible that patients underwent surgery at another clinic and for that reason, the failure rate could be underestimated.

## CONCLUSION

In this large cohort study of more than 2000 patients, the overall failure rate of all‐inside meniscal repair was 20.2%. Repair without concomitant ACLR, medial meniscal repair, and older age were associated with an increased hazard of failure. These findings underscore the clinical importance of recognising patient‐specific risk factors for meniscal repair failure, as such knowledge can guide surgical decision‐making and improve individualised treatment strategies to enhance long‐term knee function.

## AUTHOR CONTRIBUTIONS

**Christoffer von Essen**: Conceptualisation; data processing; statistical analysis; methodology; manuscript writing. All authors: Methodology; critical review of the manuscript. All authors commented on previous versions of the manuscript. All authors read and approved the final manuscript.

## CONFLICT OF INTEREST STATEMENT

The authors declare no conflicts of interest.

## ETHICS STATEMENT

Ethical permission for this study was obtained from the Regional Ethics Committee of Karolinska Institute (Diarie number: 2024‐06048‐01).

## Data Availability

The data that support the findings of this study are available on request from the corresponding author. The data are not publicly available due to privacy or ethical restrictions.

## References

[1] Abram SGF , Judge A , Beard DJ , Wilson HA , Price AJ . Temporal trends and regional variation in the rate of arthroscopic knee surgery in England: analysis of over 1.7 million procedures between 1997 and 2017. Has practice changed in response to new evidence? Br J Sports Med. 2019;53:1533–1538. 30279217 10.1136/bjsports-2018-099414

[2] Abram SGF , Price AJ , Judge A , Beard DJ . Anterior cruciate ligament (ACL) reconstruction and meniscal repair rates have both increased in the past 20 years in England: hospital statistics from 1997 to 2017. Br J Sports Med. 2020;54:286–291. 30661013 10.1136/bjsports-2018-100195

[3] Bradley KE , Cevallos N , Jansson HL , Lansdown DA , Pandya NK , Feeley BT , et al. Younger patients are more likely to undergo arthroscopic meniscal repair and revision meniscal surgery in a large cross‐sectional cohort. Arthroscopy. 2022;38:2875–2883.e1.e2871. 35688314 10.1016/j.arthro.2022.04.020

[4] Elmallah R , Jones LC , Malloch L , Barrett GR . A meta‐analysis of arthroscopic meniscal repair: inside‐out versus outside‐in versus all‐inside techniques. J Knee Surg. 2019;32:750–757. 30130810 10.1055/s-0038-1668123

[5] Engler ID , Moradian JR , Pockros BM , Schirmeister CM , Richmond JC , Salzler MJ . Patient‐reported outcomes of meniscal repair and meniscectomy in patients 40 years of age and older show similar good results. Knee Surg Sports Traumatol Arthrosc. 2021;29:2911–2917. 33025055 10.1007/s00167-020-06299-5

[6] Everhart JS , Higgins JD , Poland SG , Abouljoud MM , Flanigan DC . Meniscal repair in patients age 40 years and older: a systematic review of 11 studies and 148 patients. Knee. 2018;25:1142–1150. 30414793 10.1016/j.knee.2018.09.009

[7] Everhart JS , Magnussen RA , Cavendish PA , Axcell K , Blackwell R , Kaeding CC , et al. Subjective knee function and risk of failure are equivalent for men and women at 5 years after meniscus repair. Arthroscopy. 2020;36:816–822. 31919022 10.1016/j.arthro.2019.09.030

[8] Farinelli L , Meena A , Sonnery‐Cottet B , Vieira TD , Pioger C , Gigante A , et al. Increased intra‐articular internal tibial rotation is associated with unstable medial meniscus ramp lesions in anterior cruciate ligament‐injured athletes. Arthrosc Sports Med Rehabil. 2024;6:100839. 38187951 10.1016/j.asmr.2023.100839 PMC10768481

[9] Gerritsen LM , van der Lelij TJN , van Schie P , Fiocco M , van Arkel ERA , Zuurmond RG , et al. Higher healing rate after meniscal repair with concomitant ACL reconstruction for tears located in vascular zone 1 compared to zone 2: a systematic review and meta‐analysis. Knee Surg Sports Traumatol Arthrosc. 2022;30:1976–1989. 35072757 10.1007/s00167-022-06862-2 PMC9165248

[10] Grant JA , Wilde J , Miller BS , Bedi A . Comparison of inside‐out and all‐inside techniques for the repair of isolated meniscal tears: a systematic review. Am J Sports Med. 2012;40:459–468. 21737837 10.1177/0363546511411701

[11] Hagmeijer MH , Kennedy NI , Tagliero AJ , Levy BA , Stuart MJ , Saris DBF , et al. Long‐term results after repair of isolated meniscal tears among patients aged 18 years and younger: an 18‐year follow‐up study. Am J Sports Med. 2019;47:799–806. 30802135 10.1177/0363546519826088

[12] Howard DH . Trends in the use of knee arthroscopy in adults. JAMA Intern Med. 2018;178:1557–1558. 30264154 10.1001/jamainternmed.2018.4175 PMC6584718

[13] Kopf S , Beaufils P , Hirschmann MT , Rotigliano N , Ollivier M , Pereira H , et al. Management of traumatic meniscus tears: the 2019 ESSKA meniscus consensus. Knee Surg Sports Traumatol Arthrosc. 2020;28:1177–1194. 32052121 10.1007/s00167-020-05847-3 PMC7148286

[14] Kurzweil PR , Tifford CD , Ignacio EM . Unsatisfactory clinical results of meniscal repair using the meniscus arrow. Arthroscopy. 2005;21:905.e1–e7. 16086560 10.1016/j.arthro.2005.06.002

[15] Lee GP , Diduch DR . Deteriorating outcomes after meniscal repair using the Meniscus Arrow in knees undergoing concurrent anterior cruciate ligament reconstruction: increased failure rate with long‐term follow‐up. Am J Sports Med. 2005;33:1138–1141. 16000655 10.1177/0363546505275348

[16] Michel PA , Domnick CJ , Raschke MJ , Hoffmann A , Kittl C , Herbst E , et al. Age‐related changes in the microvascular density of the human meniscus. Am J Sports Med. 2021;49:3544–3550. 34591716 10.1177/03635465211039865

[17] Mosich GM , Lieu V , Ebramzadeh E , Beck JJ . Operative treatment of isolated meniscus injuries in adolescent patients: a meta‐analysis and review. Sports Health Multidiscipl Appr. 2018;10:311–316. 10.1177/1941738118768201 PMC6044115 29648924

[18] Nepple JJ , Block AM , Eisenberg MT , Palumbo NE , Wright RW . Meniscal repair outcomes at greater than 5 years: a systematic review and meta‐analysis. J Bone Joint Surg. 2022;104:1311–1320. 35856932 10.2106/JBJS.21.01303

[19] Noyes FR , Chen RC , Barber‐Westin SD , Potter HG . Greater than 10‐year results of red‐white longitudinal meniscal repairs in patients 20 years of age or younger. Am J Sports Med. 2011;39:1008–1017. 21278428 10.1177/0363546510392014

[20] Orellana F , Grassi A , Hlushchuk R , Wahl P , Nuss KM , Neels A , et al. Revealing the complexity of meniscus microvasculature through 3D visualization and analysis. Sci Rep. 2024;14:10875. 38740845 10.1038/s41598-024-61497-2 PMC11091062

[21] Ow ZGW , Law MSN , Ng CH , Krych AJ , Saris DBF , Debieux P , et al. All‐cause failure rates increase with time following meniscal repair despite favorable outcomes: a systematic review and meta‐analysis. Arthroscopy. 2021;37:3518–3528. 34058318 10.1016/j.arthro.2021.05.033

[22] Ozeki N , Seil R , Krych AJ , Koga H . Surgical treatment of complex meniscus tear and disease: state of the art. J ISAKOS. 2021;6:35–45. 33833044 10.1136/jisakos-2019-000380

[23] Paxton ES , Stock MV , Brophy RH . Meniscal repair versus partial meniscectomy: a systematic review comparing reoperation rates and clinical outcomes. Arthroscopy. 2011;27:1275–1288. 21820843 10.1016/j.arthro.2011.03.088

[24] Petersen W , Karpinski K , Bierke S , Müller Rath R , Häner M . A systematic review about long‐term results after meniscus repair. Arch Orthop Trauma Surg. 2022;142:835–844. 33913009 10.1007/s00402-021-03906-z PMC8994714

[25] Poland S , Everhart JS , Kim W , Axcell K , Magnussen RA , Flanigan DC . Age of 40 years or older does not affect meniscal repair failure risk at 5 years. Arthroscopy. 2019;35:1527–1532. 31000396 10.1016/j.arthro.2018.11.061

[26] Rizvanovic D , Waldén M , Forssblad M , Stålman A . Surgeon's experience, sports participation and a concomitant MCL injury increase the use of patellar and quadriceps tendon grafts in primary ACL reconstruction: a nationwide registry study of 39,964 surgeries. Knee Surg Sports Traumatol Arthrosc. 2023;31:475–486. 35896755 10.1007/s00167-022-07057-5 PMC9898417

[27] Ronnblad E , Barenius B , Engstrom B , Eriksson K . Predictive factors for failure of meniscal repair: a retrospective dual‐center analysis of 918 consecutive cases. Orthop J Sports Med. 2020;8(3):2325967120905529. 32284936 10.1177/2325967120905529 PMC7137129

[28] Schweizer C , Hanreich C , Tscholl PM , Blatter S , Windhager R , Waldstein W . Meniscal repair outcome in 3829 patients with a minimum follow‐up from 2 years up to 5 years: a meta‐analysis on the overall failure rate and factors influencing failure. Am J Sports Med. 2024;52:822–831. 37022676 10.1177/03635465231158385

[29] Schweizer C , Hanreich C , Tscholl PM , Ristl R , Apprich S , Windhager R , et al. Nineteen percent of meniscus repairs are being revised and failures frequently occur after the second postoperative year: a systematic review and meta‐analysis with a minimum follow‐up of 5 years. Knee Surg Sports Traumatol Arthrosc. 2022;30:2267–2276. 34671817 10.1007/s00167-021-06770-x PMC9206598

[30] Sedgwick MJ , Saunders C , Getgood AMJ . Systematic review and meta‐analysis of clinical outcomes following meniscus repair in patients 40 years and older. Orthop J Sports Med. 2024;12(8):23259671241258974. 39131093 10.1177/23259671241258974 PMC11311169

[31] Shieh AK , Edmonds EW , Pennock AT . Revision meniscal surgery in children and adolescents: risk factors and mechanisms for failure and subsequent management. Am J Sports Med. 2016;44:838–843. 26818451 10.1177/0363546515623511

[32] Steadman JR , Matheny LM , Singleton SB , Johnson NS , Rodkey WG , Crespo B , et al. Meniscus suture repair: minimum 10‐year outcomes in patients younger than 40 years compared with patients 40 and older. Am J Sports Med. 2015;43:2222–2227. 26187129 10.1177/0363546515591260

[33] van der Wal WA , Meijer DT , Hoogeslag RAG , LaPrade RF . Meniscal tears, posterolateral and posteromedial corner injuries, increased coronal plane, and increased sagittal plane tibial slope all influence anterior cruciate ligament‐related knee kinematics and increase forces on the native and reconstructed anterior cruciate ligament: a systematic review of cadaveric studies. Arthroscopy. 2022;38:1664–1688. e1661. 34883197 10.1016/j.arthro.2021.11.044

[34] Villarreal‐Espinosa JB , Berreta RS , Pallone L , Rubin J , Allende F , Gómez‐Verdejo F , et al. Failure and complication rates following meniscal all‐inside and inside‐out repairs: a systematic review and meta‐analysis. Knee Surg Sports Traumatol Arthrosc. 2024;33:1992–2009. 39350499 10.1002/ksa.12485

[35] Vint H , Quartley M , Robinson JR . All‐inside versus inside‐out meniscal repair: a systematic review and meta‐analysis. Knee. 2021;28:326–337. 33482623 10.1016/j.knee.2020.12.005

[36] Wells ME , Scanaliato JP , Dunn JC , Garcia EJ . Meniscal injuries: mechanism and classification. Sports Med Arthrosc. 2021;29:154–157. 34398118 10.1097/JSA.0000000000000311

[37] Yamasaki S , Hashimoto Y , Nishida Y , Teraoka T , Terai S , Takigami J , et al. Assessment of meniscal healing status by magnetic resonance imaging t2 mapping after meniscal repair. Am J Sports Med. 2020;48:853–860. 32167835 10.1177/0363546520904680

